# Association and Interaction of Epstein–Barr Virus with SARS-CoV-2 Infection—A Review

**DOI:** 10.3390/v17070903

**Published:** 2025-06-26

**Authors:** Supriya Mahajan, Saurabh Mahajan, Sayashree Patgiri

**Affiliations:** 1 Department of Microbiology, School of Medical Sciences and Research, Sharda University, Greater Noida 201306, Uttar Pradesh, India; 2 Department of Neurosurgery, Max Superspeciality Hospital, Patparganj, New Delhi 110092, India

**Keywords:** Epstein–Barr virus, SARS-CoV-2, Long COVID, novel therapeutics

## Abstract

Despite the significant decrease in SARS-CoV-2-related mortality, COVID-19 continues to impose a high public health burden due to the high rate of post-COVID-19 pathological conditions, broadly termed Long COVID, that continue for any period of time and are generally multisystemic. However, recent studies have strengthened the evidence that the reactivation of the Epstein–Barr virus (EBV) in the post-COVID-19 era has significantly contributed to the exacerbation and prolongation of Long COVID symptoms. The mechanism and pathophysiology of EBV reactivation in Long COVID patients still need further exploration due to limited studies. This review summarises the various studies linking EBV reactivation in Long COVID along with its pathophysiology and novel therapeutics for EBV in a post-COVID-19 era.

## 1. Introduction

The Epstein–Barr virus (EBV) is a double-stranded DNA virus included in the herpes family and is also called human herpesvirus 4 (HHV-4) [1]. It is a leading cause of infectious mononucleosis and was the first human oncogenic virus to be identified, specifically in Burkitt lymphoma cells [2]. It is globally present in 90% of the population [3] and is associated with various lymphoproliferative disorders including Burkitt’s lymphoma, Hodgkin’s lymphoma, T and natural killer (NK) cell lymphomas, nasopharyngeal carcinoma, gastric adenocarcinoma, post-transplant lymphoproliferative disease and AIDS-associated lymphoblastoma [3,4].

EBV exhibits a biphasic life cycle, comprising a latent phase where the virus remains asymptomatic and a lytic phase that leads to the production of infectious virions [2]. This virus can switch from latent to lytic phases, a process known as EBV reactivation, which can be triggered by various stimuli, including psychological stress and immunosuppression [3].

Severe acute respiratory syndrome coronavirus-2 (SARS-CoV-2) caused the novel coronavirus disease 2019 (COVID-19) pandemic that produced an unprecedented burden on approximately 185 countries [5]. The pandemic further led to the development of Long COVID, which is defined as new or persistent symptoms that begin three months after a probable or confirmed SARS-CoV-2 infection and last for at least two months, with no other plausible explanation [6]. Although a number of studies have suggested the role of autoimmune factors and viral persistence in the development of Long COVID, the role of EBV in both COVID-19 and Long COVID needs to be explored. This review highlights the association of EBV with SARS-CoV-2 with special emphasis on EBV reactivation and immune dysregulation in Long COVID, EBV epidemiology and pathogenesis in COVID-19, and novel therapeutics for EBV in a post-COVID-19 era.

## 2. Mechanism of EBV Reactivation

The immediate–early (IE) genes BZLF1 (Z, Zta, ZEBRA, EB1) and BRLF1 (R, Rta) are encoded by EBV and are essential for initiating lytic reactivation [7], wherein BZLF1 has been speculated to be more important because more efficient reactivation can be achieved by exogenous expression of BZLF1 as compared to BRLF1 [8]. Reactivation occurs in the following three stages [9]:

Stage 1—BZLF1 and BRLF1 are immediately transcribed after the viral entry within host cells, causing significant upregulation in the expression of the EBV lytic phase by not only activating the viral lytic cascade but also synergistically activating their own promoters, leading to the expression of early-stage genes like DNA polymerase catalytic subunits BALF1, BMRF1, and BALF2.

Stage 2—The viral genome is replicated.

Stage 3—Late genes are expressed, and their encoded proteins form the structural components of the virus, including the capsid, viral antigens, and glycoproteins. This is followed by the assembly of the components and genome, after which the virus buds out from the host cell.

Various pathways have been implicated in the reactivation process, including the Phosphoinositide 3-kinase (PI3K) pathway, the c-Jun N-terminal Kinase (JNK) pathway, and the mitogen-activated protein kinase (MAPK) pathway [2,10]. EBV reactivation at the cellular level involves the induction of one of the proteins PI3K, MAPK, or PKC followed by the induction of the transcription factors Sp1, MEF2D, AP1, and c-jun, which bind to the BZLF1-promotor region. BZLF1 transcriptional activation, triggered by this process, marks the beginning of the EBV lytic cycle. Alternatively, a double-stranded DNA break derived from damaged mitochondria or nuclei also triggers the same pathway, leading to Sp1 induction or possibly causing an AKT activation cascade [2].

## 3. EBV Reactivation and SARS-CoV-2 Infection

EBV initially infects resting B cells, which then become a reservoir for the virus as latently infected memory B cells in the peripheral blood, expressing latent membrane protein 2 (LMP-2) and EBV nuclear antigens (EBNAs). SARS-CoV-2 infection reactivates EBV-infected B cells that express LMP-2 and EBNA-1, resulting in severe systemic inflammation [11]. Although various studies have been conducted that demonstrate EBV reactivation cases ranging from 13% to 65% among Long COVID patients [12,13,14,15,16,17,18,19,20,21,22,23], as shown in Table 1, there has been limited literature on the actual mechanism of EBV lytic reactivation in Long COVID cases. There has been little published data that depict the results of the different aspects of the still unknown mechanism. 

The various proposed mechanisms involved in EBV reactivation in Long COVID are as follows.

### 3.1. EBV Reactivation Induced by Drugs Used for COVID-19 Treatment

Recent data has indicated that certain drugs used for the treatment of COVID-19 patients, such as azithromycin, chloroquine diphosphate, hydroxychloroquine sulfate, and nafamostat mesylate [24,25], increased viral lytic gene expression via the activation of MAPK and NF-kB signalling pathways [26].

Recently, Chen J. et al. conducted a study where remdesivir; which was authorised by the United States Food and Drug Administration (FDA) for COVID-19 treatment; increased the expression of viral lytic genes, such as BZLF1 (immediate early gene) and BHFR1 (early gene), in all three EBV+ lymphoma cell lines; i.e., RPMI 6666 (Hodgkin’s lymphoma), Akata (Burkitt’s lymphoma), and VAL (diffuse large B cell lymphoma), as quantified by qRT-PCR [27]. Remdesivir also reduced STAT3 but increased p38 MAPK phosphorylation from EBV+ lymphoma cells, which are two signalling pathways that are associated with EBV reactivation [28,29]. Hence, remdesivir treatment requires the continuous monitoring of viral loads and risk assessment of developing EBV-associated malignancies, even after patients have fully recovered from COVID-19. The association of some important anti-SARS-CoV-2 drugs with EBV reactivation is shown in Table 2 [26,27,28,29,30,31].

Fluvoxamine is a selective serotonin reuptake inhibitor (SSRI) and a σ-1 receptor (S1R) agonist [32] that inhibits acid sphingomyelinase (ASM) activity and the formation of the ceramide-enriched membrane domain. It also attenuates SARS-CoV-2 cell entry and decreases SARS-CoV-2 replication as well as subsequent endoplasmic reticulum (ER) stress and inflammation [33]. S1R agonists inhibit the splicing of mRNA-encoding X-box binding protein-1 (XBP1) by preventing inositol-requiring enzyme 1α (IRE1) activity; hence reducing XBP1 activation, which modulates the ER stress response pathway and reduces cytokine storm [34]. ER stress and unfolded protein response upregulate latent membrane protein 1 (LMP1), which enhances the production of interleukin-8, thus inducing EBV lytic gene expression [35,36].

### 3.2. EBV Reactivation Induced by Host Proteins Interacting with SARS-CoV-2 Virus

Recent reports have shown the interaction between various SARS-CoV-2 proteins and host proteins. Interestingly, these host proteins listed in Table 3 [37,38,39,40,41,42,43,44,45,46,47,48,49,50,51] have been shown to play a vital role in EBV lytic reactivation or the maintenance of EBV inside cells. 

Some important host proteins linked with the SARS-CoV-2 virus and their role in EBV reactivation are explained below. 

#### 3.2.1. Role of BRD4 Host Protein in EBV Reactivation

BRD4 host protein linked to SARS-CoV-2 E-protein has been shown to interact with EBNA1 through N-terminal sequences to mediate EBV transcriptional activation. Also, BRD4 is specifically localised to the FR enhancer element regulated by EBNA1, which further enhances transcriptional activation by EBNA1 [52]. BRD4 is a member of the Bromodomain and Extra-Terminal (BET) domain proteins that uses its bromodomains to bind to acetylated histones H3 and H4 and associate with interphase chromatin and mitotic chromosomes, thus leading to cell cycle progression and the development of viral oncogenes [53]. JQ1, a BET inhibitor, prevents EBV reactivation by blocking the production of the BZLF1 protein and subsequently inhibiting the transcription of downstream lytic genes. JQ1 achieves this by interfering with the recruitment of BRD4 to the BZLF1 promoter. It binds to the recognition pocket for acetylated lysine residues of BRD4 and hence competitively inhibits the BRD4-histone binding and recruitment of transcriptional complexes to the BZLF1 promoter [54].

#### 3.2.2. Role of UPF1 Host Protein in EBV Reactivation

UPF1 is a crucial protein in the nonsense-mediated decay (NMD) pathway that mediates the degradation of BRLF1 transcripts, thus acting as a negative regulator of EBV reactivation [38]. NMD is a cellular surveillance mechanism that degrades mRNAs containing premature stop codons and prevents the production of harmful proteins [55].

#### 3.2.3. Role of Anoctamin 2 (ANO2) Host Protein in EBV Reactivation

ANO2 is an ion channel expressed in the central nervous system (CNS). ANO2 antibodies recognise a fragment of EBNA1 via the mechanism of molecular mimicry, thereby causing CNS inflammation through T cells that are reactive with the same protein and triggering EBV reactivation, particularly in patients with multiple sclerosis [40].

#### 3.2.4. Role of Insulin-Degrading Enzyme (IDE) Host Protein in EBV Reactivation

IDE has a more prominent role as a cellular receptor for varicella zoster virus (VZV) where it interacts with VZV glycoprotein E (gE), and this interaction is crucial for VZV infection and cell-to-cell spread [56]. Although the role of IDE in EBV reactivation has not yet been properly studied, the fact that it interacts with a key viral protein (gE) in a closely related virus suggests a potentially similar role in EBV reactivation. IDE, however, is known to interact with amyloid-beta peptides, which are implicated in mitochondrial dysfunction and neuroinflammation, leading to fatigue and neurological symptoms experienced by ME/CFS patients [57].

#### 3.2.5. Role of HDAC2 Host Protein in EBV Reactivation

HDAC2, a histone deacetylase, removes acetyl groups from histone proteins associated with viral promoters, thus repressing viral gene transcription. HDAC inhibitors (HDACi) block the activity of HDACs, thus increasing histone acetylation levels, potentially leading to the activation of viral genes and the initiation of the EBV lytic cycle [58].

#### 3.2.6. Role of PGE2 Host Protein in EBV Reactivation

Prostaglandin E2 (PGE2), produced by the enzyme COX-2, has a convincing positive correlation with LMP1 protein, which, in turn, can upregulate COX-2, leading to accelerated lymph node metastasis in nasopharyngeal carcinoma. COX-2 can also be upregulated by EBV latent antigen EBNA3C. PGE2 signalling acts through its receptors, EP1 and EP4, which are involved in mediating inflammation and cancer progression [45].

#### 3.2.7. Role of Eukaryotic Translation Initiation Factor 4E (eIF4E) Host Protein in EBV Reactivation

The transcription of eIF4E is stimulated by the LMP1 protein, leading to nasopharyngeal carcinoma. eIF4E is significantly reduced by knocking down LMP1 and c-Myc, thereby inhibiting tumour proliferation, migration, and invasion. EIF4H also interacts with certain viral proteins, such as BZLF1 (also known as Zta or ZEBRA), which is a key regulator of the EBV lytic cycle [47].

#### 3.2.8. Role of RIPK1 Host Protein in EBV Reactivation

RIPK1 is a protein kinase involved in both cell death and survival pathways. LMP1 increases the K48 and K63 ubiquitination of RIPK1, which leads to the activation of NF-κB, a signalling pathway that promotes cell survival and replication and shifts RIPK1 signalling away from necroptosis (cell death) [59].

### 3.3. EBV Lytic Replication Induces ACE2 Expression and Enhances SARS-CoV-2 Virus Entry

The spike protein of SARS-CoV-2 mediates viral entry into the cells by binding to the ACE2 receptor on epithelial cells. Verma D. et al. observed that EBV lytic gene expression increases ACE2 expression in EBV-infected epithelial cells. Changes in cellular gene expression after EBV lytic replication were analysed and a 500-fold increase in ACE2 mRNA levels was observed 24 h after lytic induction, which remained elevated for two days. Additionally, Verma D. et al. also observed that the ACE2 promoter further enhances EBV lytic expression as it contains response elements for Zta, an EBV transcriptional activator, which preferentially acts on methylated promoters, allowing them to reactivate epigenetically silenced EBV promoters from latency, hence leading to EBV entry into the lytic cycle of replication. Furthermore, SARS-CoV-2 virus entry into the cells was measured using a luciferase assay and it was found that EBV replication led to a 5--to-6-fold increase in SARS-CoV-2 entry. This indicates that subclinical EBV replication and lytic EBV gene expression may enhance the efficiency and extent of SARS-CoV-2 infection in humans [60].

### 3.4. Other Hypothetical Proposed Mechanisms of EBV Reactivation

#### 3.4.1. Trogocytosis-Induced EBV Reactivation

Human primary B lymphocytes are the major reservoirs for EBV, and it seems quite reasonable that SARS-CoV-2 infection can trigger EBV reactivation in EBV-positive B cells. However, the irony here is that the SARS-CoV-2 virus attaches to the ACE2 receptor, which is not expressed well in B cells [61]. This irony has been adequately solved through a biological phenomenon known as trogocytosis, wherein cells share membrane-associated proteins, viral receptors, and viral particles during cell–cell conjugation. Previous studies have demonstrated that NK cells acquired a receptor for EBV from EBV-infected B cells [62], dendritic cells acquired HIV-1 from infected T cells [63], and B cells acquired membrane-bound α2,3 sialic acid receptor molecules from monocytes via trogocytosis [64]. Although direct studies related to SARS-CoV-2 are lacking, it can be speculated that EBV-infected B cells might be able to steal an ACE2 receptor via trogocytosis and then become susceptible to SARS-CoV2 infection.

#### 3.4.2. Exosome-Mediated EBV Reactivation

The second possible explanation of EBV reactivation during Long COVID is based on the role of exosomes that are usually released by a host cell during the course of any viral infection, thus carrying viral and host components that can trigger an immune response. Barberis E. et al. performed a proteomic analysis of plasma exosomes and found the presence of SARS-CoV-2 RNA in host exosomal cargo using a reverse transcription–droplet digital polymerase chain reaction (RT–ddPCR), which can be speculated to have helped SARS-CoV-2 in spreading cell-to-cell infection via an endocytosis route [65]. Similarly, it can be speculated that exosomes containing SARS-CoV-2 RNA can spread infection from healthy B cells to B cells already infected with EBV.

## 4. Inflammation—A Common Thread Binding EBV and COVID-19

**Figure 1:**
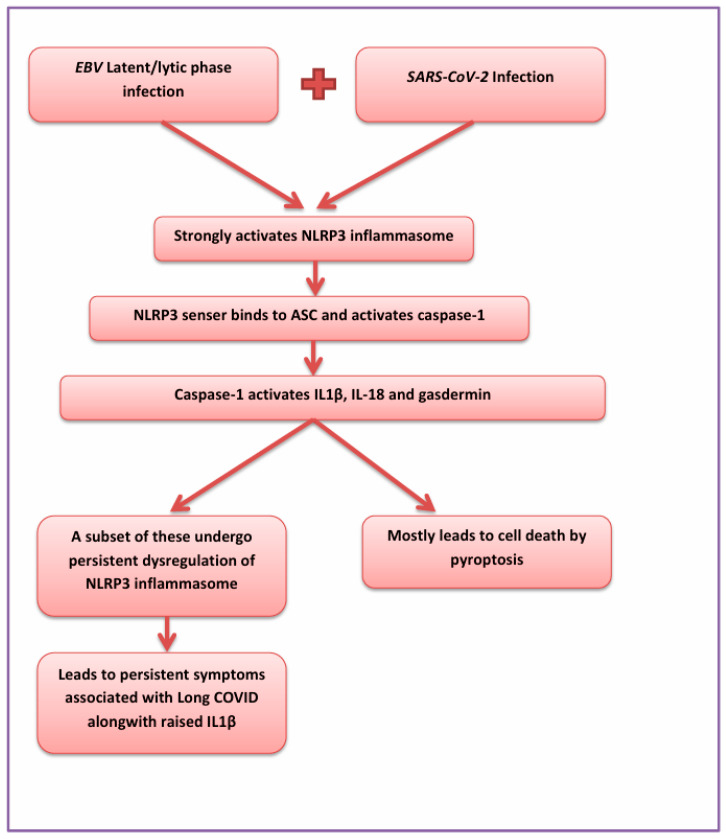
Flowchart showing inflammatory cascade associated with EBV and SARS-CoV-2 co-infection.

SARS-CoV-2 is known to cause the activation of the NLRP3 inflammasome [66,67], which in turn stimulates EBV reactivation [68]. Recent studies have demonstrated that EBV promotes the production of the inflammatory cytokines interleukin (IL)-1β and tumour necrosis factor-α (TNF-α) as well as the chemokines IL-8 and monocyte chemoattractant protein-1 (MCP-1) through the TLR9–MyD88–NF-κB pathway, which are also major cytokines implicated in Long COVID and other inflammatory diseases, hence linking EBV, inflammation, and COVID-19 [69]. Figure 1 depicts the inflammatory cascade associated with the co-infection of EBV and SARS-CoV-2 infection, leading to persistent symptoms of Long COVID.

An inflammatory state is further maintained by the following actions of innate and adaptive immune cells [69]:

(a) There is an increase in neutrophils that maintain an inflammatory state by producing cytokines.
(b) Macrophages become coated with myelin and adopt a foamy morphology. Foamy macrophages can maintain inflammation via EBV exosomes, cytokine production, and recruitment of more macrophages.
(c) Monocytes maintain inflammation by carrying viral infection long after the initial infection has been resolved.
(d) Inflammation is maintained by higher levels of IFN-γ and TNF-α, producing SARS-CoV-2-specific CD4+ and CD8+ T cells.
(e) Autoantibodies produced by B cells contribute to tissue damage.

Also, monocyte-derived macrophages exposed to EBV antigen-laden exosomes express more CXCL10, which is a pro-inflammatory chemokine that attracts monocytes, eosinophils, T cells, and NK cells [70]. Therefore, it can be hypothesised that EBV antigen-laden exosomes can induce further inflammation [69].

SARS-CoV-2 infection induces a severe acute hyperinflammatory shock, termed multisystem inflammatory syndrome (MIS-C), in children and adolescents at four to eight weeks after infection [71]. Acute MIS-C is characterised by the impaired reactivation of SARS-CoV-2-reactive memory T cells, which is accompanied by the presence of TGFβ in T cells, B cells, and monocytes, along with the reduced antigen-presentation capabilities of monocytes, and can be reversed by blocking TGFβ. Goetzke C.C. et al. demonstrated that T cell receptor repertoires of patients with MIS-C exhibit an expansion of T cells expressing TCRVβ21.3, resembling EBV-reactive T cell clones; hence, serum TGFβ in patients with MIS-C can trigger EBV reactivation, which is reversible with TGFβ blockade [72].

## 5. EBV Reactivation Contributing to COVID-19-Associated Autoimmunity

Another common feature shared between SARS-CoV2 and EBV is the development or exacerbation of autoimmune phenomena. Although EBV has been associated with rheumatoid arthritis for the last 40 years [73], there has been a renewed interest regarding its pathogenetic role in several autoimmune diseases [74], particularly rheumatoid arthritis [75] and multiple sclerosis [76]. It has been hypothesised that EBV may increase the risk of developing autoimmunity in COVID-19 patients due to its ability to disrupt B cell tolerance [77]. Various studies have shown a high frequency of EBV reactivation in COVID-19 patients with severe illness and those suffering from Long COVID symptoms, including autoimmune diseases [16,17,19,20]. SARS-CoV-2 triggers the production of cytokine storm and causes the dysregulation of innate immune and adaptive immune responses with hyper-activation of immune system cells, and all of which lead to worsening of autoimmune diseases as well as Long COVID manifestations. The reactivation of latent EBV is favoured by COVID-19-induced immune dysregulation in the host. EBV can cause or exacerbate autoimmunity through different mechanisms, like the hyper-activation of autoreactive B cells, plasma cell differentiation, autoantibody production, tissue damage, and bystander activation. COVID-19-related immunological changes are exacerbated by EBV reactivation, which may elevate the likelihood of developing Long COVID complications [77].

## 6. Novel Therapeutics for EBV and Their Efficacy Against SARS-CoV-2

Various novel drugs and drugs under investigation have been used against EBV, some of which have also shown considerable potency against the SARS-CoV-2 virus, as shown in Table 4 [78,79,80,81,82,83,84,85,86,87,88,89,90,91,92]. 

Some of the novel drugs for EBV are as follows.

### 6.1. Nucleoside Analogues (Acyclovir, Valacyclovir, Ganciclovir, and Valganciclovir)

Acyclovir is an important acyclic nucleoside that is more readily phosphorylated by viral thymidine kinases than the corresponding cellular thymidine kinases, with an inherent flexibility that allows for optimised interactions in target enzyme binding sites. However, it has low bioavailability; due to this, a valine ester prodrug of acyclovir, (valacyclovir) was introduced, which had a 3-to-5-fold increase in oral bioavailability that led to an improved efficacy against herpesviruses. Gancyclovir is an acyclic guanosine mimic that retains the 3′-hydroxyl group. It also demonstrates low bioavailability, which can be increased with the addition of a valine ester to yield the prodrug valganciclovir [93].

Hocker B. et al. evaluated the efficacy of ganciclovir and valganciclovir prophylaxis on EBV viral load in EBV-naïve pediatric renal transplant recipients (R−) who had received a graft from an EBV-positive donor (D+) and found a significant decrease in the EBV primary infection [94].

Another study by Østensen A.B. et al. on pediatric liver transplant recipients showed that there was no difference in the proportion of patients with a reduction in virus load in patients treated with ganciclovir compared to untreated patients at 8 weeks [95].

### 6.2. Nucleotide Analogues (Cidofovir)

Cidofovir is a nucleoside analogue of deoxycytidine monophosphate primarily approved for the treatment of cytomegalovirus (CMV) retinitis in AIDS patients. It inhibits viral DNA synthesis by acting as a competitive inhibitor and alternative substrate for CMV DNA polymerase, blocking further viral DNA synthesis through its incorporation into the growing DNA strand. Cidofovir is also proven to be effective against other DNA viruses, including EBV [96]. Abdulkarim B. et al. demonstrated that cidofovir can significantly reduce LMP1 and EBNA2 mRNA and protein expression, along with the enhancement of radiation-induced apoptosis and radiosensitivity in EBV-related malignancies via the proteolytic cleavage of death effectorscaspase-9 and -3 [83].

### 6.3. Pyrophosphate Analogues (Foscarnet)

Foscarnet inhibits herpesvirus DNA polymerases by blocking the pyrophosphate-binding site and preventing the cleavage of pyrophosphate from deoxynucleoside triphosphates. Although approved for the treatment of CMV retinitis in AIDS patients, it has potentially shown activity against all human herpesviruses, including EBV [92], as shown in Table 4.

### 6.4. Fluvoxamine

Fluvoxamine is a selective serotonin reuptake inhibitor and sigma-1 receptor agonist that is primarily used to treat obsessive–compulsive disorder (OCD) but has also shown activity against SARS-CoV-2 via a reduction in platelet aggregation, decreased mast cell degranulation, the regulation of inositol-requiring enzyme 1α-driven inflammation, and interference with endolysosomal viral trafficking [97]. It also attenuates SARS-CoV-2 cell entry by inhibiting acid sphingomyelinase (ASM) activity and the formation of a ceramide-enriched membrane domain [33]. Fluvoxamine reduces XBP1 activation, which in turn modulates the ER stress response pathway and reduces cytokine storm. XBP1 plays a major role in EBV reactivation and hence its deactivation by fluvoxamine helps to prevent lytic gene expression in EBV [33].

### 6.5. Maribavir

Maribavir is an oral benzimidazole riboside approved by the U.S. Food and Drug Administration in 2021 for the treatment of post-transplant cytomegalovirus infection that is refractory to treatment with ganciclovir, valganciclovir, cidofovir, or foscarnet. It inhibits viral protein kinase UL97, which further inhibits the phosphorylation of the UL44 protein, thus inhibiting CMV DNA replication [98]. It has shown in vitro activity against EBV by inhibiting both viral DNA replication and viral transcription [90] along with the inhibition of BGLF4, which largely affects EBV transcript levels [99].

### 6.6. KAY-2-41 and KAH-39-149

KAY-2-41 (1′-methyl-substituted 4′-thiothymidine) and KAH-39-149 (4′-azido analogue of 4′-thiothymidine) are thiothymidine derivatives that have shown in vitro inhibitory activities against EBV. It has been observed that mutations in the viral thymidine kinase (TK) of KAY-2-41- and KAH-39-149-resistant herpesviruses conferred only low-level resistance to these drugs but high-level resistance to other TK-dependent antiviral agents. Coen et al. found that the antiviral efficacy of KAH-39-149 was superior to KAY-2-41 in a mouse model of gammaherpesvirus infection [79].

### 6.7. Brincidofovir (CMX-001)

Brincidofovir is a prodrug of cidofovir that releases cidofovir intracellularly, allowing for higher intracellular and lower plasma concentrations of cidofovir, effectively increasing its oral bioavailability and activity against dsDNA viruses. But despite its enhanced antiviral activity, it is not yet FDA-approved for adenovirus or CMV due to a lack of efficacy in clinical trials [91].

### 6.8. Inhibitors of EBV Nuclear Antigen 1 (EBNA1)

EBNA1 inhibitors decrease the expression of EBNA1, which is involved in the maintenance, replication, and segregation of the EBV genome. Lee E.K. et al. characterised H31 as an EBNA1 inhibitor, which inhibits EBNA1-dependent oriP sequence-specific DNA binding activity and produces the gradual loss of EBV episome, thereby delaying the growth of EBV-infected lymphoblastoid cell lines or Burkitt’s lymphoma cells [100].

### 6.9. Newer Drugs Used to Reduce Inflammation

#### 6.9.1. Ibrutinib

Ibrutinib, a Bruton tyrosine kinase (BTK) inhibitor, has shown promise in treating EBV-related lymphoproliferative disorders, particularly in immunocompromised patients. Kotaki R. et al. demonstrated that Ibrutinib inhibits the survival of EBV-positive lymphoblastic cell lines (LCLs) and Aggressive NK cell leukemia (ANKL) cells in vitro [101]. This drug also reduces the ability of NLRP3 to reduce IL1β, thereby reducing inflammation [102].

#### 6.9.2. MCC950

MCC950, a selective NLRP3 inflammasome inhibitor, plays a role in potentially treating EBV reactivation by blocking the activation of the NLRP3 inflammasome via the inhibition of its ATP hydrolysis activity and the subsequent release of inflammatory cytokines like IL-1β and IL-18. This small molecule has not yet progressed beyond the Phase I safety trial. It has good potential for preventing the development of EBV-related cancers by limiting EBV-induced B cell transformation [103].

## 7. Conclusions

There is sufficient evidence regarding the contribution of COVID-19 in EBV reactivation from the latent to lytic phases, leading to a worsening of Long COVID manifestations. Recent studies have hypothesised various mechanisms leading to EBV reactivation but the data is severely limited. Data on post-COVID-19 EBV reactivation in the context of autoimmune conditions is also limited, making it difficult to establish a direct role for EBV in the development or worsening of these conditions following SARS-CoV-2 infection. The boundary between the immunological effects of SARS-CoV-2 alone and the effects of latent co-infecting viruses, like EBV, still remains unclear and deserves further investigation.

## Figures and Tables

**Table 1:** Studies reporting EBV reactivation in patients with COVID-19.

S. No.	Type of Study	Study Population	Study Findings	Reference
1.	Systematic review	53 articles and 40 reactivation studies	58% of COVID-19 patients presented signs of EBV reactivation	[12]
2.	Systematic review	19 studies; 539 patients who were infected with both COVID-19 and Herpesviridae	EBV reactivation was frequent in COVID-19 patients; D-dimer, C-reactive protein (CRP), length of stay in the intensive care unit (ICU), and usage of invasive mechanical ventilation were significantly associated markers	[13]
3.	Retrospective study	106 patients; 54 positive and 52 negative for COVID-19	27.1% of EBV reactivations, based on qPCR detection of EBV genomes, were from the COVID-19-positive group, while only 12.5% of reactivations belonged to the negative group	[14]
4.	Retrospective study	67 COVID-19 patients with onset time within 2 weeks	55.2% of patients were seropositive for EBV viral capsid antigen (VCA) IgM antibodies. EBV/SARS-CoV-2 coinfection was associated with fever and increased inflammation	[15]
5.	Retrospective study	68 COVID-19 patients	66.7% of Long COVID subjects versus 10% of control subjects were positive for EBV reactivation based on positive titers for EBV early antigen-diffuse (EA-D) IgG or EBV VCA IgM	[16]
6.	Retrospective study	117 ICU patients with severe COVID-19	16% of patients with severe COVID-19 developed EBV reactivation as compared to 14% in the non-COVID-19 control group	[17]
7.	Retrospective study	104 COVID-19 patients, including 42 hospitalised in ICU and 62 in a sub-ICU	EBV DNA median level among ICU patients was significantly higher than that observed in SICU patients, and the B cell count was significantly increased in ICU patients	[18]
8.	Retrospective study	128 COVID-19 patients	13.3% COVID-19 patients demonstrated EBV reactivation. Lymphocyte and albumin of EBV group decreased more significantly than the non-EBV group. Respiratory failure, acute respiratory distress syndrome (ARDS), and hypoproteinaemia of EBV group had more incidence than non-EBV group	[19]
9.	Observational retrospective cohort study	120 patients with COVID-19 severe pneumonia were enrolled from ICU	EBV reactivation was observed in 65% of patients, with authors claiming that viral reactivation is associated with mortality and a higher risk of developing both ventilator-associated pneumonia (VAP) and ICU bloodstream infections (BSI)	[20]
10.	Longitudinal study	309 COVID-19 patients from initial diagnosis to convalescence (2–3 months later)	EBV reactivation in COVID-19 patients at the time of COVID-19 diagnosis was positively correlated with Long COVID symptoms of fatigue and sputum production 2 to 3 months after COVID-19 onset	[21]
11.	Monocentric retrospective study	34 COVID-19 patients from ICU were selected	EBV was detected in 82% of patients with EBV reactivation occurring early after ICU admission and was associated with longer ICU length-of-stay	[22]
12.	Pilot observational study	88 patients with post-COVID-19 manifestations were recruited	Patients with post-COVID manifestations presented with reactivation of EBV in 42.6% of cases, with authors claiming that patients with herpes virus infections presented with more frequent fever, headache, psycho–neurological disorders, pulmonary abnormalities, myalgia, activation of liver enzymes, elevated CRP and D-dimers, and suppressed cellular immune response	[23]

**Table 2:** Association of anti-SARS-CoV-2 drugs with EBV reactivation.

S. No.	Anti-SARS-CoV-2 Drugs	Role in EBV Reactivation
1.	Remdesivir	○ Increases the expression of viral lytic genes—BZLF1 (immediate early gene) and BHFR1 (early gene) [27]. ○ Reduces STAT3 but increases p38 MAPK phosphorylation from EBV+ lymphoma cells [28,29].
2.	Azithromycin and nafamostat mesylate	○ Significantly increase viral lytic gene expression and virion production via the activation of MAPK and NF-kB signalling pathways [26].
3.	Chloroquine diphosphate	○ Triggers EBV replication through phosphorylation of Kruppel-associated and Box-associated protein 1/tripartite motif-containing protein 28 (KAP1/TRIM28) in Burkitt lymphoma cells [30].
4.	Dexamethasone	○ Leads to dose-dependent upregulation of immediate early gene BZLF1 mRNA expression that encodes the lytic transactivator protein ZEBRA [31].

**Table 3:** Role of interactive host proteins linked to their respective SARS-CoV-2 proteins in EBV reactivation.

S. No.	SARS-CoV-2 Protein	Interactive Host Proteins Associated with EBV Reactivation	Role in EBV Reactivation
1.	E	BRD4; BRD2	Activate EBV enhancer and promoter function to modulate gene expression. JQ1 blocks BZLF1 protein production and prevents downstream transcription of lytic genes [37].
2.	N	UPF1; DDX21	UPF1 knockdown upregulates the expression of EBV lytic genes BZLF1, BMRF1, BLLF1, and BcLF1 and elevates lytic proteins ZEBRA and EA-R [38]. DDX21 inhibits EBV lytic replication by interacting with EBV protein SM [39].
3.	M	ANO2; FAKD5; MPPA	Anoctamin 2 (ANO2) exhibits molecular mimicry with EBV nuclear antigen 1 and increases the risk of multiple sclerosis [40]. FAKD5 and MPPA affect the remodelling of EBV-infected B cells [41].
5.	NSP2	RAP1GDS1	Undergoes downregulation in the presence of EBNA1, which causes latent viral replication in B cells [42].
6.	NSP4	IDE	Major protein modulated by EBV protein deoxyuridine triphosphate nucleotidohydrolase (dUTPase), which is associated with neuroinflammation observed in myalgic encephalomyelitis/chronic fatigue syndrome (ME/CFS) [43].
7.	NSP5	HDAC2	Involved in histone deacetylase inhibitor (HDACi)-induced EBV reactivation [44].
9.	NSP7	PGES2	PGES2 produces PGE2, which is a principle COX-2-regulated downstream product. Upregulation of COX-2 modulates EBV lytic reactivation through its downstream effector PGE2 [45].
10.	NSP8	EXOSC3	Included among the major genes modulated by EBV-encoded dUTPase in human dendritic cells [46].
11.	NSP9	EIF4H	LMP1 stimulates the transcription of eIF4E via c-Myc to promote EBV-mediated nasopharyngeal carcinoma [47].
12.	NSP12	RIPK1; TCF12	LMP1 can modulate the post-translational modification of receptor-integrating protein kinase 1 (RIPK1) and LMP1-mediated K63-polyubiquitination of RIPK1, which can induce a switch from necroptotic death to survival [48]. LMP2 promoter binds to TCF12, blocking reactivation of EBV [49].
13.	ORF3a	SUN2	SUN2 is a nuclear envelope-associated protein that is modified by BCLF4 protein during EBV reactivation and is required for further EBV replication [50].
14.	ORF10	CUL2	CUL2 forms complexes with EBV ZEBRA proteins and induces p53 degradation for promoting EBV propagation [51].

**Table 4:** Novel therapeutics for EBV and their efficacy against SARS-CoV-2.

S. No.	Novel Therapeutics	Comments
1.	Nucleoside Analogues (acyclovir, valacyclovir, ganciclovir, and valganciclovir)	○ Studies have shown that acyclovir and ganciclovir inhibit EBV in vitro [78,79]. ○ Meng M. et al. showed that ganciclovir-treated patients had an improved survival rate in comparison to patients with COVID-19 who did not receive ganciclovir therapy [80]. ○ German E.R. et al. showed that acyclovir resolved the neurological symptoms of COVID-19 patients and lowered their IgG and IgM titers, supporting the use of acyclovir for COVID-19 neurologic symptoms [81].
2.	Nucleotide Analogues (cidofovir)	○ Successful treatment of recurrent EBV-associated nasopharyngeal carcinoma with cidofovir was reported in two patients [82]. ○ Cidofovir enhanced radiation-induced apoptosis and radiosensitivity in EBV-related malignancies (Burkitt’s lymphoma and nasopharyngeal carcinoma) [83]. ○ In a study by Rehman et al., an ADMET (Absorption, Distribution, Metabolism, Excretion, and Toxicity) analysis revealed that cidofovir is well metabolised, distributed, and bioavailable, but has some undesirable effects [84].
3.	Pyrophosphate Analogues (foscarnet)	○ Foscarnet in combination with immunoglobulins was successful in controlling persistent EBV infection in a lung transplant patient that showed clinical improvement of Post-Transplant Lymphoproliferative Disorder (PTLD) following a reduction in immunosuppression intensity [85]. ○ Schneider U. et al. reported that two AIDS patients with EBV-associated lymphoproliferative disorders who did not respond to standard chemotherapy achieved complete tumour regression after therapy with foscarnet [86].
Anti-EBV Compounds Under Investigation		
a.	Fluvoxamine	○ Considering the interaction of XBP1 and sigma-1 receptor in the ER, it is likely that sigma-1 receptor agonists (i.e., fluvoxamine), which may block EBV reactivation, would be potential therapeutic drugs to limit clinical deterioration after infection and Long COVID symptoms [87,88].
b.	Maribavir (MBV)	○ Oral benzimidazole L-riboside with significant activity against both Human Cytomegalovirus (HCMV) and EBV but no other human herpesviruses [89]. ○ MBV has been shown to inhibit the phosphorylation of the EBV DNA polymerase processivity factor BMRF1 [63] and has a unique dual effect against the EBV inhibition of viral DNA replication and virus transcription [90].
c.	KAY-2-41 and KAH-39-149	○ These two thionucleoside derivatives were effective in vitro anti-EBV activity [79].
d.	Brincidofovir (CMX-001)	○ This alkoxyalkyl ester prodrug of cidofovir has the same in vitro broad-spectrum antiviral activity as cidofovir but with an activity up to 1000-fold higher compared with cidofovir due to higher intracellular levels of cidofovir-diphosphate [91].
e.	Inhibitors of EBV Nuclear Antigen 1 (EBNA1)	○ The EBV-encoded nuclear antigen 1 (EBNA1) is a versatile protein useful in the maintenance, replication, and segregation of EBV genome and represents an attractive therapeutic target to treat EBV-associated malignancies [92].
